# Tailoring Freeze-Drying for Starter Cultures Preservation: A Case Study with *Lactiplantibacillus plantarum*

**DOI:** 10.3390/foods15111928

**Published:** 2026-05-29

**Authors:** Antoni Miecznikowski, Katarzyna Wierzchowska, Renata Choińska, Agata Fabiszewska

**Affiliations:** 1 Department of Fermentation Technology, Prof. Wacław Dąbrowski Institute of Agricultural and Food Biotechnology—State Research Institute, Rakowiecka 36 Street, 02-532 Warsaw, Polandrenata.choinska@ibprs.pl (R.C.); 2 Department of Chemistry, Institute of Food Sciences, Warsaw University of Life Sciences, Nowoursynowska 159c, 02-776 Warsaw, Poland; katarzyna_wierzchowska@sggw.edu.pl

**Keywords:** freeze drying, lactic acid bacteria, cryoprotectants

## Abstract

Freeze-drying is a crucial technique for preserving bacterial strains, yet its efficiency depends heavily on the precise selection of protective agents. This study aimed to optimize freeze-drying conditions for the commercially relevant, non-GMO strain *Lactiplantibacillus plantarum* K KKP/593/p. The cryoprotective effects of glycerol, dimethyl sulfoxide (DMSO), and trehalose were evaluated, alongside various carriers including skim milk powder, maltodextrin, inulin, and starch. Survival rates were determined using the plate count method on MRS agar, complemented by scanning electron microscopy (SEM) for microstructural analysis. Results indicated that skim milk powder was the superior carrier, significantly outperforming polysaccharides. Among the protective agents, glycerol exhibited the highest efficacy, while trehalose and DMSO were suboptimal. The most effective formulation 20% glycerol without prior incubation combined with skim milk powder at 0.75:1 (*w*/*w*, total mass) ratio maintained maximum viability with no statistically significant decrease. SEM observations confirmed that this synergistic combination ensured a stable, porous matrix favorable for rehydration. These findings emphasize that while synergistic multi-component systems are essential for maximizing post-process viability, cryoprotective formulations must be empirically tailored to specific bacterial strains to ensure industrial efficiency.

## 1. Introduction

Microorganisms play a crucial role in the global biotechnology industry due to their wide range of applications. From a technological perspective, it is essential to ensure the stability and reproducibility of microbial biomass and the products obtained from it. Therefore, the development and optimization of preservation methods remain an important area of research [1,2,3,4].

Among the available preservation methods, freeze-drying (lyophilization) is one of the most effective techniques for long-term storage of microorganisms, including bacteria, yeasts, and fungi [4,5,6]. The process is based on the removal of water by sublimation, which reduces biochemical activity and allows microorganisms to remain in reversible anabiosis while maintaining their technological properties [7].

Lyophilization, in addition to long-term stability, offers a number of advantages, such as protection against contamination, relatively high survival rates, and convenient storage and distribution. However, the process is also associated with significant cell damage and reduced viability [2,8]. During freezing and drying, cells are exposed to multiple stress factors, including ice crystal formation, osmotic stress, dehydration, and pH changes, which can damage cell membranes, proteins, and nucleic acids [2,9,10]. Recent studies emphasize that cell damage should be considered as a cumulative effect of all stages of the process rather than a single-step phenomenon [3,11].

Process parameters play a critical role in determining cell survival. Freezing conditions, in particular, influence ice crystal formation and osmotic stress, while factors such as cell density, physiological state, and rehydration conditions further affect survival rates [6,9,12]. To enhance microbial survival, protective agents are commonly added before freeze-drying. Their mechanisms of action include stabilizing cell membranes, reducing dehydration-induced damage, and forming glassy matrices that preserve biomolecular structures. However, the effectiveness of protective agents is strongly dependent on the specific microbial strain and process conditions. The selection of an effective cryoprotectant is not merely empirical but is deeply rooted in the physicochemical stabilization of the system. Key factors, such as the glass transition temperature (Tg) of the amorphous matrix and the final water activity (aw) of the lyophilizate, determine the long-term stability of the bacteria. A high Tg is essential to maintain the product in a glassy state, thereby restricting molecular mobility and preventing the deleterious effects of matrix collapse and oxidative stress. Furthermore, controlling water activity is crucial, as even minor fluctuations in residual moisture can significantly impact membrane integrity and protein stability during storage [3,9,13]. Understanding these mechanistic aspects is vital for developing formulations that provide a robust protective barrier against the stresses of sublimation and subsequent shelf life.

Lactic acid bacteria (LAB) represent a diverse group of microorganisms widely used in the food and feed industries as starter cultures and probiotics [14]. Despite their technological importance, LAB are relatively sensitive to environmental stresses, including dehydration and temperature fluctuations, which makes their preservation particularly challenging [1,4]. Although freeze-drying is considered the most effective preservation method for LAB, survival rates remain highly variable and strongly strain-dependent. This variability highlights the need for strain-specific optimization strategies, as also emphasized in recent studies on probiotic stability and formulation [6,15]. Despite numerous studies on microbial freeze-drying, there is still no universal formulation ensuring high survival of LAB across different strains. This indicates the need for further research to optimize the composition of protective systems and process parameters in a strain-specific manner.

This study aimed to determine the effects of selected carriers and protective agents on the survival of freeze-dried *Lpb. plantarum* cells and, consequently, to select the most effective combination of additives under specific freeze-drying conditions. The focus was placed on a non-genetically modified strain with a long-standing history of successful commercial application in the production of high-quality silage for animal feed and fermented food products. Despite the widespread use of lyophilization in biotechnology, ensuring the long-term viability of high-density bacterial preparations remains a critical challenge for maintaining their functional and metabolic performance. Ultimately, this study provides a practical solution to enhance the industrial efficiency of the commercially proven strain *Lpb. plantarum* K KKP/593/p in both the agricultural and fermented food sectors. The novelty of this approach lies in its comprehensive optimization of the synergy between protein-based carriers and polyols, enabling a robust, scalable formulation for stable, high-quality probiotic preparations. To complement the results, images obtained with a scanning electron microscope were examined.

## 2. Materials and Methods

### 2.1. Materials

The material used in the study was the lactic acid bacteria strain *Lactiplantibacillus plantarum* K KKP/593/p, obtained from the Collection of Industrial Microbial Cultures of the Prof. Wacław Dąbrowski Institute of Agricultural and Food Biotechnology—National Research Institute (Warsaw, Poland). The strain is characterized by high lactic acid production efficiency and the ability to ferment a wide range of carbohydrates, including amylolytic activity, as confirmed by Polish patent no. 179838. Bacterial cultures were stored in glycerol stocks at −20 °C. The following carriers were used: inulin (Hortimex, Konin, Poland), soluble potato starch (Avantor Performance Materials Poland S.A., Gliwice, Poland), cold-soluble starch (Zetpezet, Piła, Poland), maltodextrin (Pepees S.A., Łomża, Poland), skim milk powder (Spółdzielnia Mleczarska, Gostyń, Poland), calcium gluconate (Polfarmex, Kutno, Poland), sodium caseinate (Kazeina Polska Sp. z o.o., Legionowo, Poland), and sorbitol (Merck Life Science Sp. z o.o., Poznań, Poland). The cryoprotectants included glycerol (Avantor Performance Materials Poland S.A., Gliwice, Poland), dimethyl sulfoxide (DMSO) (Avantor Performance Materials Poland S.A., Gliwice, Poland), and trehalose (Hayashibara, Okayama, Japan).

### 2.2. Biomass Cultivation

Bacterial cultivation was performed in a Biostat B2 fermenter (Sartorius, Göttingen, Germany) with a total volume of 10 L for 24 h at 35 °C and pH 5.8, regulated with 12.5% ammonia solution. The cultivation was carried out under microaerophilic conditions. The culture medium consisted of sucrose (Diamant, Poznań, Poland), bactopeptone and peptone (Becton, Dickinson and Company, Franklin Lakes, NJ, USA), yeast extract (Biomed, Lublin, Poland), corn steep liquor (Polfa Tarchomin, Warsaw, Poland), malt extract (Wytwórnia Ekstraktów Słodowych, Wolsztyn, Poland), and mineral salts: diammonium hydrogen phosphate (Chempur, Piekary Śląskie, Poland), ammonium sulfate, magnesium sulfate, and manganese sulfate (POCH, Poland). After cultivation, biomass was separated using a centrifuge (Beckman J2-MC, Beckman Coulter, Brea, CA, USA) at 9500 rpm for 15 min, washed with saline solution, and re-centrifuged under the same conditions.

### 2.3. Lyophilization Process

The freeze-drying process was conducted using a Christ Alpha 1–4 freeze-dryer (B. Christ, Osterode am Harz, Germany). Samples were placed on metal trays (77 mm diameter) and frozen directly in the lyophilizer chamber in a controlled manner with an average cooling rate ranging from 0.22 °C/min to 0.38 °C/min. The condenser temperature was maintained at −55 °C, and the chamber pressure during primary and secondary drying was reduced to below 0.1 mbar (10 Pa). The total process time, including the sublimation and final drying phases, ranged from 38 to 43 h, depending on the cryoprotectant variant. The ambient temperature during the process was stable at 20 ± 2 °C. After completion of secondary drying, the system was gradually returned to atmospheric pressure. The samples were then removed and stored in sealed containers until the ambient temperature was reached to prevent moisture absorption.

### 2.4. Experimental Design

**Figure 1:**
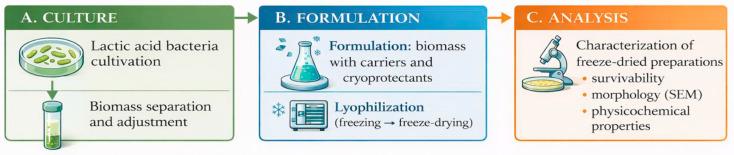
Schematic diagram of the experimental workflow.

Biomass was mixed with selected carriers and/or cryoprotectants in defined proportions prior to lyophilization. The dry matter content was adjusted to approximately 15% using a saline solution. Carrier-to-biomass ratios ranged from 0.5:1 to 0.75:1 (*w*/*w*, total mass). The selection of carrier-to-biomass ratios and cryoprotectant concentrations was based on preliminary screening experiments and on established literature values for lactic acid bacteria. Selected carriers (inulin, maltodextrin, soluble starch, and skim milk powder) were further analyzed. Cryoprotectants (glycerol, DMSO, and trehalose) were applied at concentrations ranging from 2.5% to 40%, with the best concentration at 15%. In selected experimental variants, samples were incubated with cryoprotectants for 4 h prior to freeze-drying. The experimental design aimed to optimize formulation composition to maximize bacterial survivability. All experiments were conducted in triplicate as independent biological replicates. For each experimental series, a fresh batch of bacterial biomass was prepared, and a separate control sample (biomass lyophilized without protective agents) was included. Consequently, the survival rates of the control samples ranged from 32.3% to 39.2% across the different series. A schematic representation of the experimental procedure is presented in Figure 1.

### 2.5. Microbiological and Analytical Methods

Bacterial counts were determined using plate count methods on MRS agar, followed by incubation at 37 °C for 48 h. Survivability (%) was calculated as the ratio of viable cell counts before and after lyophilization. Dry matter content was determined using a moisture analyzer (Sartorius, Germany).

The microstructure of selected samples was analyzed using scanning electron microscopy (SEM, FEI Quanta 200, FEI Company, Hillsboro, OR, USA). The samples were mounted on aluminum stubs using double-sided carbon adhesive tape. Observations were carried out at an accelerating voltage of 20 kV and a working distance (WD) of approximately 10 mm. Images were captured using a secondary electron (SE) detector at various magnifications (from 300× to 3000×). The analysis focused on evaluating matrix porosity, the presence of bacterial cells embedded in the carrier, and the overall homogeneity of the lyophilizate.

### 2.6. Statistical Analysis

Data were expressed as mean ± standard deviation. Statistical analysis was performed using one-way ANOVA, treating each protective formulation as an independent experimental variant to facilitate direct comparison between specific cryoprotective recipes. Homogeneity of variance and normality were verified using Bartlett’s, Levene’s, and Shapiro–Wilk tests. Differences between means were assessed using Tukey’s post hoc test at a significance level of *p* < 0.05. All analyses were performed using Statistica 8 software (StatSoft Inc., USA).

## 3. Results

### 3.1. Selection of the Carrier

**Figure 2:**
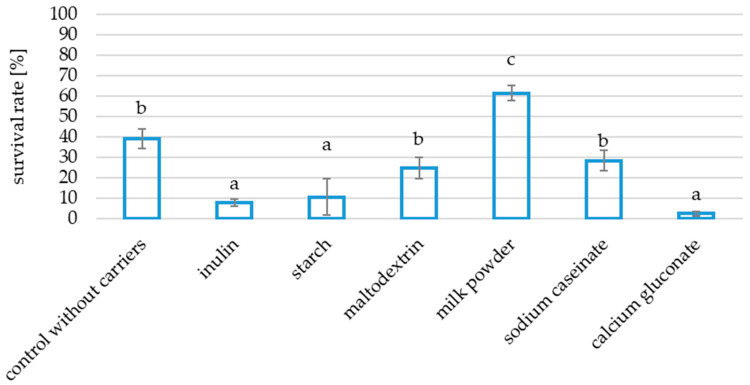
Survival rates of *Lpb. plantarum* K KKP/593/p for groups of preparations containing various carriers (inulin, starch, maltodextrin, skimmed milk powder, sodium caseinate, and calcium gluconate). Values with different letters are significantly different (*p* < 0.05) according to Tukey’s test.

In the initial phase of the study, the survival rates of bacteria lyophilized with and without the addition of six different carriers were evaluated. Based on the obtained results, skim milk powder, maltodextrin, soluble potato starch, and inulin were selected for further analysis. Although inulin showed a lower survival rate in the initial phase of the study, the survival rates of bacteria lyophilized with and without the addition of six different carriers were evaluated. Of the materials tested, skim milk powder provided the most effective cryoprotection, resulting in the highest bacterial viability of (61.4%) (Figure 2). Preparations containing maltodextrin (24.6%) and soluble potato starch (10.5%) yielded survival rates lower than those of the control group (39.2%), yet remaining within a range considered suitable for further optimization. In contrast, the use of sodium caseinate (28.4%), inulin (7.6%), and calcium gluconate (2.4%) resulted in a substantial decrease in viability compared with the control.

The physical properties of the preparations also varied significantly depending on the carrier. Maltodextrin demonstrated the best technological performance, offering the most efficient mixing with biomass and excellent rehydratability, resulting in a stable, homogeneous suspension. Although skim milk powder presented some challenges during the grinding process due to its texture, it ensured proper rehydration and a uniform suspension. Inulin and soluble potato starch also allowed for efficient grinding and satisfactory rehydration. Conversely, preparations containing calcium gluconate and sodium caseinate were remarkably hard, making them difficult to pulverize. Furthermore, their rehydration was problematic, and achieving a homogeneous suspension was impossible.

Based on these results, its excellent processing characteristics (ease of grinding and rehydration) justified its inclusion in the next phase of optimization. Calcium gluconate and sodium caseinate were excluded from further studies due to their poor protective properties and unfavorable physical characteristics, particularly their excessive hardness and failure to form uniform suspensions. Despite inulin’s moderate cryoprotective performance in the initial screening, it was included in the subsequent optimization phase due to its well-known prebiotic properties and its role as a bulking agent. The aim was to evaluate whether combining it with other carriers could yield a synergistic effect, enhancing both the functional value of the starter and the physical structure of the final lyophilizate.

### 3.2. Selection of a Protective Agent

**Figure 3:**
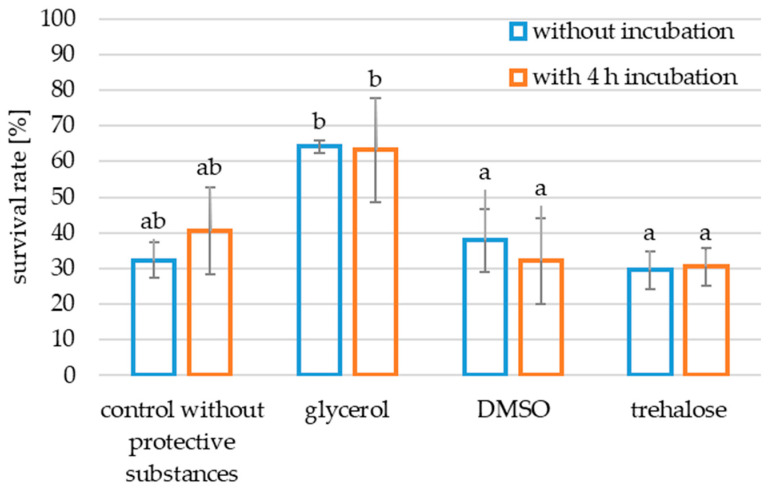
Survival of freeze-dried *Lpb. plantarum* K KKP/593/p preparations using various preservatives, without incubation, and with 4-h incubation with a preservative. Values with different letters are significantly different (*p* < 0.05) according to Tukey’s test.

The second stage of the study evaluated the cryoprotective efficacy of glycerol, dimethyl sulfoxide (DMSO), and trehalose, both with and without a 4 h incubation period. The results of the bacterial survival rates are summarized in Figure 3.

In the variants without incubation, glycerol proved to be the most effective cryoprotectant, showing bacterial survival up to 64.2%, compared to 32.3% in the control. This result suggested a positive numerical trend in improving cryoprotective potential; however, the difference was not statistically significant, as both variants belonged to the same homogeneous statistical group. The optimal glycerol concentration was found to be 20% relative to the dry biomass weight. In contrast, DMSO was used at lower concentrations; exceeding a 15% dosage led to a rapid decline in survival. Trehalose did not exhibit a protective effect, with survival rates (29.6%) comparable to or lower than those of the control group. Statistical analysis confirmed these differences, distinguishing two homogeneous groups: one with higher survival (glycerol and control) and another with lower viability (DMSO, trehalose, and control).

The introduction of a 4-h incubation at room temperature prior to lyophilization did not significantly alter the overall efficiency ranking of the tested substances, but it did affect the stability of the results (Figure 3). Glycerol maintained its superior protective capacity, with a survival rate of 63.2%. Optimal performance was observed at intermediate concentrations (10–20%). DMSO showed a slight protective effect at low concentrations, but higher dosages significantly impaired bacterial viability, resulting in a mean survival of 32.1% (with concentrations above 20% being particularly detrimental). Trehalose remained ineffective across concentrations, yielding a mean survival of 30.5%, which was lower than that of the incubated control (40.5%). The statistical analysis for the incubated variants again identified two homogeneous groups. The group with the highest survival was represented by the glycerol-supplemented preparations and the control, while the DMSO and trehalose treatments showed significantly lower viability, failing to outperform the control.

**Figure 4:**
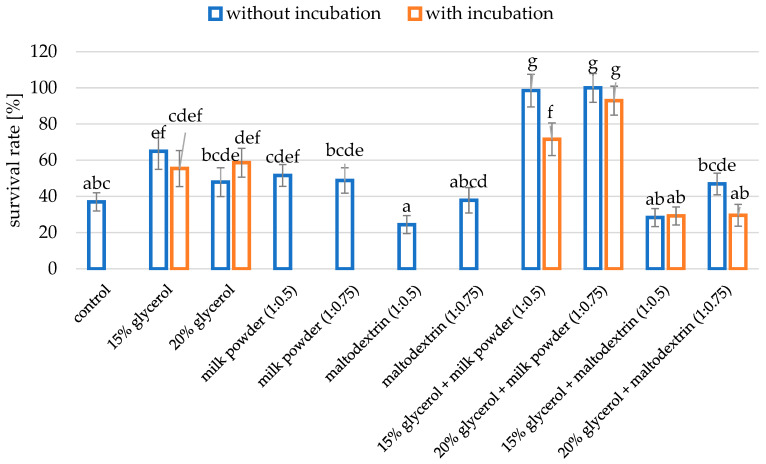
Survival of freeze-dried *Lpb. plantarum* K KKP/593/p preparations using various preservatives at different concentrations and carriers in different ratios in relation to biomass. Values with different letters are significantly different (*p* < 0.05) according to Tukey’s test.

In the initial screening phase, the addition of glycerol alone did not result in a statistically significant improvement in survival compared to the control (*p* > 0.05), placing them in the same homogeneous group. However, a clear upward trend in viability was observed. The protective effect was fully realized only when glycerol was combined with skim milk, resulting in the highest survival rate (Figure 4).

### 3.3. Selecting the Combination of Carrier and Protective Agent

In the final stage of the preliminary optimization, the combined effect of the most effective protective substance, glycerol (at 15% and 20%), and selected carriers, including skim milk powder and maltodextrin (at ratios of 0.5:1 and 0.75:1 (*w*/*w*, total mass)), was evaluated. The initial bacterial count was 4.80 × 10^11^ CFU/g. The results, expressed as survival rates (Figure 4), revealed a remarkable synergistic effect between glycerol and skim milk powder. The highest viability was achieved for the preparation containing 20% glycerol and skim milk powder at a 0.75:1 ratio, without incubation, resulting in a total survival rate of 100%. Similarly, the variant with 15% glycerol and milk powder at a 0.5:1 ratio without incubation yielded a high survival rate of 98.5%.

The experimental data further indicated that preparations containing only skim milk powder, with survival rates of 51.6% and 48.8%, significantly outperformed those containing maltodextrin. Notably, any addition of glycerol to milk-based preparations resulted in a substantial increase in bacterial protection compared to the pure biomass control, which showed 37.0% survival. In contrast, maltodextrin proved ineffective as a carrier in this experimental setup, with survival rates in maltodextrin-only preparations ranging from 24.4% to 37.9%. Furthermore, its combination with glycerol did not yield the expected synergistic improvement, with survival values remaining significantly lower than those observed for the milk-glycerol systems. Regarding the impact of the incubation period, it was observed that for the most effective combinations of glycerol and skim milk powder, the absence of incubation consistently resulted in higher survival rates compared to the four-hour incubation variants (100% vs. 92.9% and 98.5% vs. 71.6%, respectively). This suggests that for this specific bacterial strain, immediate stabilization of the cell structures prior to freezing is more effective than a prolonged equilibration period with the protective agents.

### 3.4. A Comparison of the Appearance of the Preparations Before and After Freeze-Drying

In preparations containing only protective agents (glycerol, DMSO, trehalose), no differences were observed prior to freeze-drying. Observation of preparations containing protective substances after freeze-drying revealed the crystallization process. Differences in the appearance of the preparations can be observed, which may result from both the composition of the preparation and its placement within the freeze-dryer chamber. In the case of preparations with added carriers: inulin, soluble potato starch, maltodextrin, and skim milk powder, depending on the level of the additive, differences in the color and consistency of the preparations can be observed. Differences were also observed after freeze-drying; primarily, as the carrier characterized by greater hardness, a lighter color, and less visible crystallization, preparations were characterized by greater hardness, a lighter color, and less visible crystallization.

**Figure 5:**
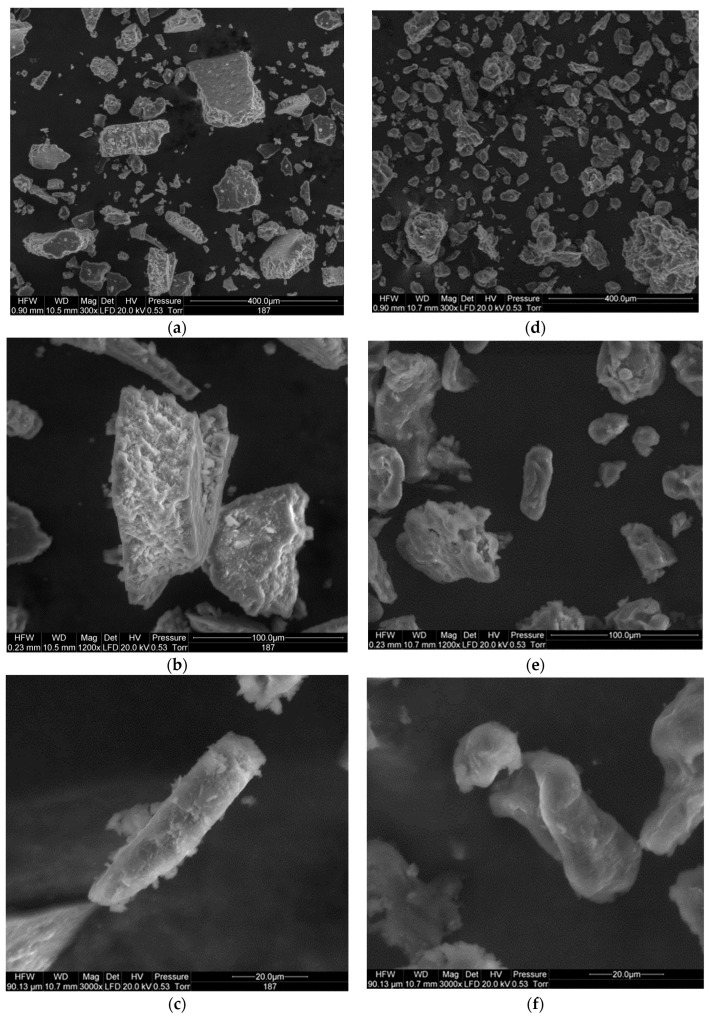
Scanning electron microscope images of a *Lpb. plantarum* bacterial biomass, without additives, (**a**–**c**) following freeze-drying and (**d**–**f**) following air-drying; (**a**,**d**)—at 300× magnification, (**b**,**e**)—at 1200× magnification, (**c**,**f**)—at 3000× magnification.

**Figure 6:**
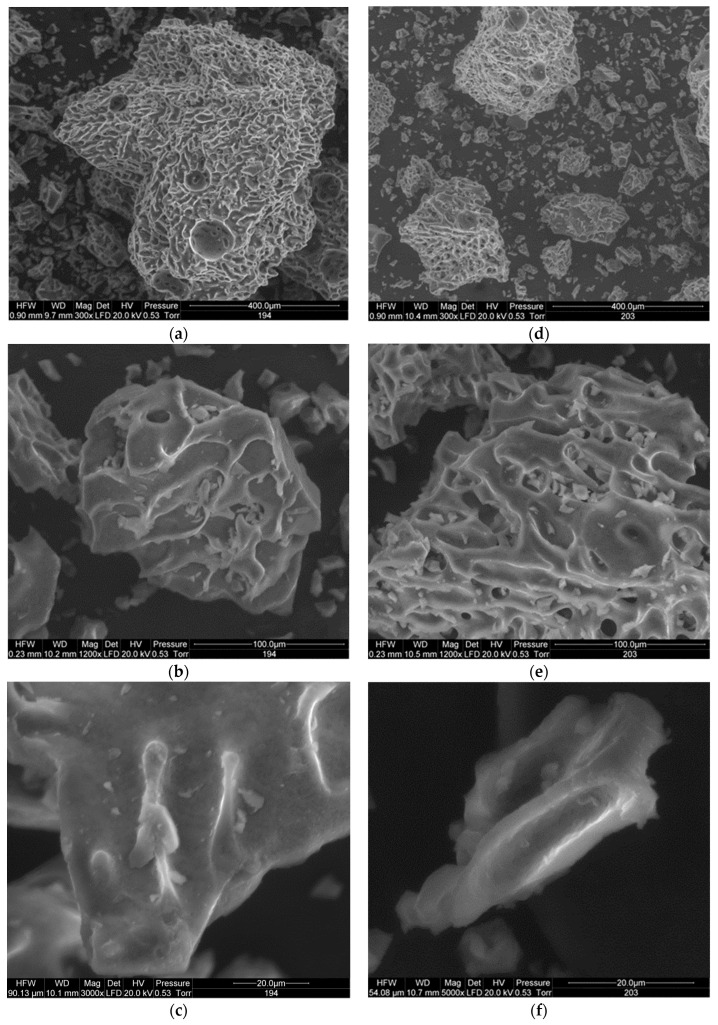
Scanning electron microscope images of a *Lpb. plantarum* K KKP/593/p bacterial biomass with the addition of maltodextrin (**a**–**c**) and 20% glycerol and maltodextrin (**d**–**f**), following freeze-drying; (**a**,**d**)—at 300× magnification, (**b**,**e**)—at 1200× magnification, (**c**,**f**)—at 3000× magnification.

**Figure 7:**
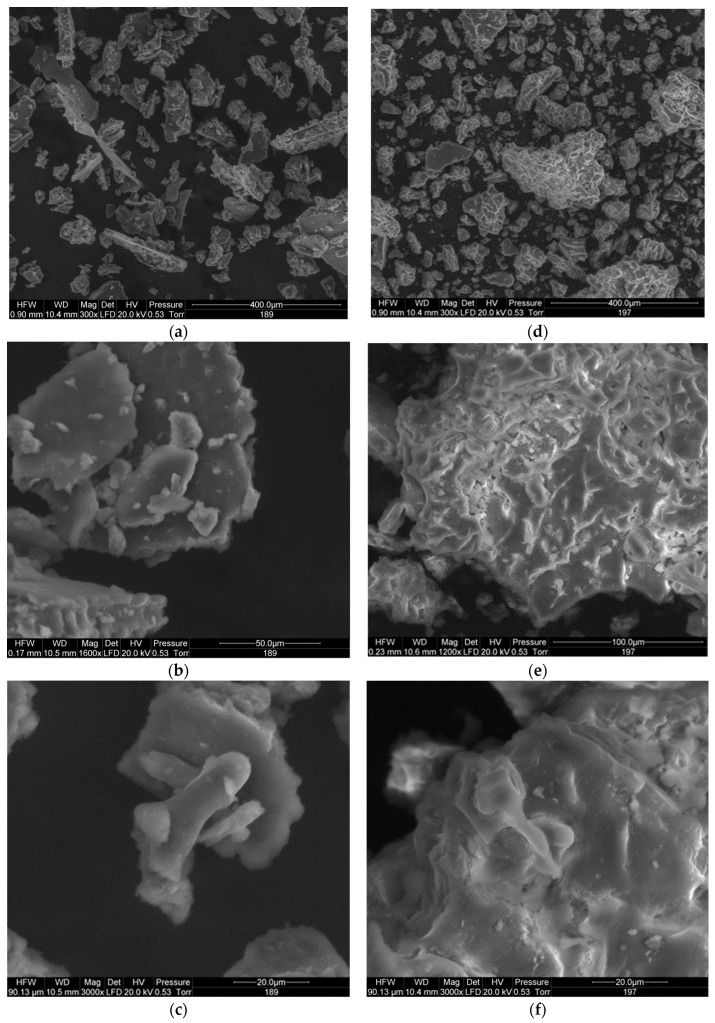
Images from a scanning electron microscope of a *Lpb. plantarum* K KKP/593/p bacterial biomass following freeze-drying with the addition of 20% glycerol without incubation (**a**–**c**) and 20% glycerol and skim milk powder, (**a**,**d**)—at 300× magnification, (**b**,**e**)—at 1600× magnification, (**c**,**f**)—at 3000× magnification.

The microstructure of selected samples from the experiment was examined using a scanning electron microscope. Photographs of selected microscopic images are shown in Figure 5, Figure 6 and Figure 7. To enable comparison of the samples’ structures, uniform magnifications were used, allowing high-resolution images to be obtained. However, these magnifications did not allow individual bacterial cells to be distinguished; they merely enabled an assessment of the structure or surface of the ground product’s granules. The genus *Lactobacillus* exhibits a high degree of morphological diversity. Depending on the composition of the culture medium and the age of the culture, these bacteria may form long, narrow, and straight rods, as well as curved forms, and even short and thick ones. Their dimensions range from 0.5 to 1.2 μm. The average length of a bacterial cell is 1–5 μm, and the diameter is 1–2 μm. *Lpb. plantarum* is a species of non-motile rod with a diameter of 0.9–1.2 μm and a length of 3–8 μm. It should be noted, however, that the SEM analysis performed in this study was strictly qualitative and descriptive; no quantitative image analysis, such as automated porosity measurements, particle size analysis, or direct bacterial distribution quantification, was conducted.

The images shown illustrate significant differences in the structure of the freeze-dried samples obtained during the experiment and, by extension, document the differing results obtained for the various test variants. A biomass sample without additives, in which bacterial survival was 37%, was presented as a reference sample (Figure 5a–c). To compare the freeze-drying process with conventional drying, a preparation dried by air-blasting with atmospheric air is also presented (Figure 5d–f). Figure 6a–c shows the preparation that achieved the lowest survival rate—24.4%—containing an addition of maltodextrin. The preparation that achieved the highest survival rate—100%—contained a 20% glycerol additive without incubation and skim milk powder (Figure 7d–f). In addition, a preparation containing 20% glycerol after incubation and maltodextrin (survival rate—29.6%) (Figure 6d–f) and a formulation with a 20% glycerol supplement without incubation (survival rate—47.9%) (Figure 7a–c).

The preparation of freeze-dried *Lpb. plantarum* without additives, when viewed under a scanning electron microscope, was characterised by the presence of clusters with sharp edges (Figure 5). Preparations containing added maltodextrin and maltodextrin combined with glycerol were characterised by a smoother structure and the formation of larger clusters (Figure 6). In turn, freeze-dried preparations composed of glycerol and skim milk also formed aggregates, but with a more granular structure than the preparations with maltodextrin (Figure 7). The preparation containing only glycerol resembled the preparation without additives in structure, but was characterised by lower granularity and the presence of smaller clusters (Figure 7). In the case of the air-dried preparation, aggregates were also observed, but they were significantly smaller in size. Furthermore, the edges of these aggregates were less sharp (Figure 5).

The observed microstructure of the skim milk–glycerol system, characterized by a highly porous and interconnected network, appears to be a key factor in the high survival rates of *Lpb. plantarum*. The sponge-like consistency of the matrix (Figure 6 and Figure 7) may suggest a relatively homogeneous incorporation of bacteria within the protective framework, potentially contributing to reduced mechanical damage during ice crystal sublimation. However, as individual bacterial cells could not be directly distinguished in the micrographs, this microstructural interpretation remains tentative and describes the general morphology of the supportive matrix rather than direct cellular distribution. Furthermore, this open porosity increases the specific surface area of the lyophilizate, which, while purely descriptive in this study, is known to facilitate the rapid and even penetration of the rehydration medium. This potentially minimizes osmotic shock during reconstitution, thereby contributing to the near 100% recovery of viable cells observed in this formulation. While these qualitative observations suggest a favorable structure for rehydration, further quantitative studies of rehydration kinetics are required to fully characterize the powder’s physical properties.

## 4. Discussion

### 4.1. The Effect of the Carrier on the Freeze-Drying Process of LAB Preparation

Among the tested carriers, skim milk powder proved to be the most effective in both improving survival and enhancing technological properties. In the initial phase, at a 0.5:1 ratio to biomass, survival reached 61.4% (compared to 39.2% in the control). In subsequent stages, a 0.75:1 ratio yielded the highest viability of 66.5%. Reddy et al. [16] also confirmed the protective effect of skim milk powder (20 g/100 mL) on *Lpb. plantarum*, achieving survival near 100%, whereas the control (water) reached only 52%. In contrast, Zayed and Roos [17] reported significantly lower survival for *Lig. salivarius* (22.4% with 18% milk), while their control survival was only 4%. These discrepancies, along with the results of Berner and Viernstein [18] (25–40% survival), may stem from differences in cell concentration (10^9^ CFU/mL), prolonged drying times (24–48 h), and varied rehydration conditions. The high efficacy of milk is attributed to its proteins and carbohydrates, which stabilize the cell membrane and protect cell wall proteins. Additionally, calcium ions in milk enhance bacterial resistance to sublimation [17], while the resulting porous structure facilitates rapid rehydration [18].

Maltodextrin and other polysaccharides are commonly used as cryoprotectants in freeze-drying because they can form a protective matrix around probiotics. For example, maltodextrin improved the survival rates of *Lcb. casei* during spray-drying and storage, maintaining microbial viability above 70% for 21 days at room temperature [19]. Although maltodextrin is known for its water-binding capacity, its high molecular weight may prevent it from interacting effectively with the polar groups of the cell membrane [20]. Polysaccharides primarily act as physical barriers and do not directly interact with bacterial membrane phospholipids. This limits their ability to stabilize membrane integrity under freeze-drying stress [21,22]. In this study, maltodextrin did not enhance survival compared to the control, yielding 24.6% viability. These findings differ from Reddy et al. [16], who reported 94% survival for *Lpb. plantarum* using maltodextrin. However, our results align with those of Castro et al. [23], where survival reached only 7% for *Lb. bulgaricus*.

Soluble potato starch also showed limited efficacy. At a 0.5:1 ratio, survival was 10.5%, and further increases in starch concentration led to a decrease in viability. Similar observations were made by Abadias et al. [5] in studies on *Candida sake*, where 10% starch yielded 11.9% survival, significantly lower than the milk-based control (22%). This confirms that starch-based matrices are generally less effective against protective agents than milk proteins.

Inulin did not provide adequate protection for *Lpb. plantarum* K KKP/593/p, with survival dropping to 7.6%. Despite its confirmed prebiotic properties and use in synbiotic formulations [24], it proved insufficient as a primary cryoprotectant in this study. Beyond the low survival rates, both sodium caseinate and calcium gluconate were excluded due to their poor technological performance. The resulting preparations were remarkably hard, making pulverization difficult, and failed to form a homogeneous suspension upon rehydration, a critical requirement for commercial applications. All presented results confirm that for the investigated *Lpb. plantarum* strain, skim milk powder is the most reliable carrier for maintaining post-process viability and optimal physical characteristics.

### 4.2. Analysis of the Selection of a Protective Substance

Cryoprotective mechanism on cells and biomolecules is, in the majority of cases, based on vitrification and glassy state formation, water replacement mechanism [25] or membrane stabilization [26]. Glycerol is widely recognized as a slow-penetrating cryoprotectant that typically requires incubation before lyophilization. It has been shown to enhance the survival of various microorganisms, including LAB, when used in optimal concentrations [27]. In the present study, the survival rates did not differ significantly between the variants with and without a four-hour incubation. In the non-incubated variant, 20% glycerol yielded 64.2% survival, while the incubated variant reached a comparable 63.2%. Still, these results confirm the high protective capacity of glycerol relative to the control. Similar effectiveness was observed by Kanmani et al. [28], who reported 89.1% survival for *Enterococcus faecium* with 35% glycerol, and by Castro et al. [29], who noted a significant increase in *Lb. bulgaricus* viability, although their survival values (17%) were lower than those presented in this work.

The protective mechanism of glycerol, as a sugar alcohol, is linked to its hydroxyl groups, which interact with the polar groups of phospholipid bilayers. Glycerol is known to reduce the crystallization rate and render the process reversible. However, it should be noted that excessive concentrations can lower the optimal freezing rate for bacteria [20,27], which may explain why survival slightly decreased at the highest tested concentration (25%).

In this study, DMSO provided only a modest increase in survival at low concentrations and led to a rapid decline in viability at higher dosages. Without incubation, the highest survival (46.2%) was observed at a 10% concentration, while concentrations exceeding 15% were detrimental. With incubation, the optimum shifted to 5% (48.2%), but higher dosages significantly impaired viability. The limited use of DMSO in lyophilization compared to freezing is likely due to its potential toxicity. DMSO increases intracellular concentration to limit ice formation, but it can become toxic at temperatures above 10 °C or during prolonged contact [30,31]. These factors likely contributed to the low survival rates and the significant drop in viability observed at higher concentrations and during incubation. While DMSO has been widely used for its cryoprotective properties, its cytotoxicity and impact on membrane integrity, especially at higher concentrations and temperatures, have led to its replacement by safer alternatives like NADESs in food-grade bacterial lyophilization. These alternatives provide effective cryoprotection with minimal toxicity, aligning with the industry’s focus on safety and sustainability [31,32].

Surprisingly, trehalose did not exhibit a protective effect in this study, with survival rates (29.6–30.5%) remaining lower than or comparable to the control group. Although trehalose is known to protect cells by replacing water molecules in hydrogen bonds with phospholipids, its poor performance in this study may be explained by its crystallization during sublimation. According to Carvalho et al. [1], crystallized trehalose loses its ability to interact with membrane proteins, thus failing to preserve the functional conformation of the biostructures during drying. This stands in contrast to several studies, such as those by Zayed and Roos [17], where trehalose significantly enhanced the viability of various lactic acid bacteria, reaching survival rates as high as 95%. However, the lack of effectiveness observed in our study is not entirely without precedent. Carvalho et al. [33] noted that trehalose was less effective than other carbohydrates for this species. The protective mechanism of trehalose involves forming glassy solids to limit ice formation and interacting with the lipid bilayer to lower its phase transition temperature [16]. Crystallization of trehalose during sublimation can minimize its ability to form hydrogen bonds with proteins, rendering it unable to protect bacterial cells during the drying process [1]. This phenomenon, likely combined with strain-specific sensitivity, may explain the poor performance of trehalose in our experimental setup. The cryoprotective effect of trehalose is highly strain-dependent. For example, in one study, *Lacticaseibacillus rhamnosus GG* showed improved survival with trehalose, but other strains like *Lcb. paracasei* and *Lcb. casei/paracasei* demonstrated varying levels of viability depending on the cryoprotectant used (e.g., skim milk, lactose, or trehalose) and storage conditions [34,35]. Not surprisingly, sucrose and skim milk often outperformed trehalose in certain conditions. For example, sucrose provided better protection than trehalose for *Lpb. plantarum* and *Lcb. rhamnosus GG* during freeze-drying [36].

The suboptimal performance of trehalose may be linked to its specific phase behavior during the rapid cooling rates applied in this study. In the absence of other stabilizing polymers, trehalose may undergo partial crystallization rather than forming a stable amorphous glass. Such crystallization prevents the water-replacement mechanism. Furthermore, the high moisture content in certain trehalose variants could have lowered the glass transition temperature, promoting molecular mobility and further destabilizing the bacterial cells during the primary drying stage.

Interestingly, our results showed that, for the most effective formulations, omitting the 4-h equilibration period led to higher survival rates. This finding partially contradicts the literature, which suggests that prolonged incubation allows cryoprotectants like glycerol to better penetrate the cell membrane, thereby providing improved protection. We hypothesize that in the case of *Lpb. plantarum* K KKP/593/p, the 4-h incubation at room temperature might have induced excessive osmotic stress before the freezing step began. Glycerol, while protective, is an osmotically active substance; prolonged exposure prior to freezing could lead to partial dehydration or alterations in membrane fluidity, rendering cells more susceptible to mechanical damage during ice crystal formation. Furthermore, it is possible that immediate freezing ‘traps’ the cells in a favorable physiological state, preventing metabolic shifts that could occur during room temperature.

### 4.3. Analysis of the Selection of Combinations of Active Ingredients and Carriers

The highest survival rates for *Lpb. plantarum* K KKP/593/p were achieved by combining glycerol (at both 15% and 20%) with skim milk powder, reaching values close to 100%. Significant improvements in viability were also observed for these combinations after a four-hour incubation period, although the non-incubated variants consistently yielded satisfying results. In contrast, preparations based on maltodextrin and glycerol were notably less effective; only the combination of 20% glycerol and maltodextrin (0.75:1 ratio) surpassed the survival rate of the pure biomass control.

The strategy of combining protective agents is well-supported by literature, as multi-component matrices often provide superior stabilization compared to single-additive systems. The promising efficacy of the skim milk and glycerol combination can be attributed to the formation of a glassy state (vitrification). As noted by Morgan et al. [2], such an amorphous matrix increases the effective viscosity around the cells, minimizing molecular mobility and preventing the formation of lethal eutectic salts. Furthermore, the proteins in milk provide a protective coating, while glycerol dissolves salts that do not crystallize with water, maintaining osmotic balance during the critical stages of freezing. Skim milk is widely recognized as an effective cryoprotectant for LAB, providing structural and osmotic protection, e.g., for *Enterococcus* sp. (more than 90% survival rate) [29]. Studies suggest that glycerol and skim milk may have interactive or synergistic effects. For example, skim milk provides a matrix that stabilizes cells, while glycerol prevents ice damage. Together, they can enhance the survival of LAB during freeze-drying or freezing [27,37,38].

For instance, Zayed and Roos [17] reported that supplementing 18% skim milk with 4% trehalose or sucrose significantly increased the viability of *Lig. salivarius* (up to 85%) compared to milk-only treatments (22%). The addition of specific protective agents to a milk-based carrier likely enhances cellular integrity by providing amino and secondary hydroxyl groups that stabilize cells during both the drying process and subsequent storage [39].

The poor performance of maltodextrin in combination with glycerol can be explained by the molecular structure of the additives. Dianawati et al. [20] noted that smaller molecules, such as three-carbon glycerol, are often more effective at protecting bacterial cells than long-chain polysaccharides like starch or maltodextrin [37]. This suggests that the larger molecular size of maltodextrin may interfere with the protective interactions of glycerol at the membrane level [20]. Ultimately, the significant variations in survival observed in this study emphasize that cryoprotection is highly strain-specific. While the technical parameters of the lyophilization process, such as freezing rates and equipment, play a role, the selection of a compatible carrier and protective agent remains the most decisive factor for maintaining the biological activity and technological properties of this specific *Lpb. plantarum* strain.

The observed synergy between skim milk and glycerol represents a significant enhancement over traditional single-component protective systems. While skim milk is a well-established carrier providing a physical matrix and buffering capacity, its combination with high concentrations of glycerol (20%) appears to offer improved protection against both osmotic and thermal stress during the sublimation phase. In many previous studies, the use of polyols or sugars alone typically resulted in survival rates ranging from 60% to 85% for *Lpb. plantarum* strains. Our findings demonstrate that the specific magnitude of improvement achieved through this protein–polyol synergy, reaching nearly 100% viability, surpasses these benchmarks. This suggests that the protein matrix of skim milk may provide a stabilizing framework that enhances the cryoprotective efficacy of glycerol, potentially through more effective hydrogen bonding and better stabilization of the cell membrane’s phospholipid bilayer.

Although the immediate survival rate reached nearly 100% for certain variants, it is important to note that the long-term viability of freeze-dried starters is closely linked to residual moisture. In the present study, water activity and residual moisture were not directly measured. Nevertheless, the process conditions employed—specifically the extended drying time and low chamber pressure—were designed to achieve the low moisture levels (typically below 3%) necessary for bacterial stabilization. Future research should incorporate water activity monitoring to further validate the storage potential of the developed formulations.

Regarding the industrial applicability of the developed formulation, the scalability of the 20% glycerol and skim milk system (0.75:1 ratio) merits further consideration. From an economic perspective, both skim milk and glycerol are cost-effective and widely approved food-grade ingredients. However, the high concentration of glycerol may pose technical challenges in large-scale production, such as increasing the hygroscopicity of the final powder and potentially lowering the glass transition temperature, which requires stringent humidity control during packaging. Nevertheless, given the high survival rates achieved, this formulation offers a robust baseline for high-value starter cultures where maximum viability is prioritized over high-throughput processing. Future pilot-scale studies should focus on optimizing the drying kinetics and evaluating the sensory impact of these cryoprotectants on the final food products to fully validate their commercial potential.

## 5. Conclusions

The freeze-drying process is a method that ensures high survival rates of the *Lpb. plantarum* K KKP/593/p bacterial strain, provided that the process parameters are optimized and selected on a case-by-case basis. The use of appropriate protective substances can significantly improve the results obtained. However, it should be noted that the high survival rates reported here are subject to experimental variability inherent in microbiological assays, and the lack of a universal formulation remains a challenge. A comparison of the results of this study with data from the literature confirms that the effect of protective agents is strain-dependent; even within a single species, differences in survival rates can be observed following the use of a given protective agent, and the type and level of the additive must be determined experimentally. The use of appropriate carriers may also improve survival rates during the freeze-drying process; an equally important role of carriers is to ensure the proper structure of moist preparations and the desired physical properties (e.g., ease of grinding, solubility) after drying. The cryoprotection of *Lpb. plantarum* has been studied extensively, but significant gaps remain in understanding strain-specific responses, optimizing cryoprotectant formulations, and ensuring long-term stability. While this study identified highly effective recipes, the conclusions are limited by the focus on immediate post-process survival rather than long-term storage stability. Furthermore, the experimental design used in this study represents a preliminary empirical optimization rather than a fully factorial optimization strategy. Consequently, further research utilizing multifactorial approaches is needed to comprehensively evaluate the complex interactions among all variables (carrier type, cryoprotectant concentration, and ratio), thereby enabling the development of a fully optimized and mathematically validated cryoprotection protocol. What is more, while SEM provided valuable insights into the matrix morphology, future research should incorporate complementary imaging techniques, such as confocal laser scanning microscopy (CLSM), to more precisely evaluate the spatial distribution and structural integrity of bacterial cells within the protective biopolymeric carrier. Addressing these gaps and integrating thermal analysis (e.g., glass transition temperature) could enhance the viability and functionality of *Lpb. plantarum* in industrial and probiotic applications.

## Data Availability

The original contributions presented in the study are included in the article; further inquiries can be directed to the corresponding author.
